# Sensing Technologies and Physiological Parameters for Real-Time Driver Drowsiness Detection: A Comprehensive Review

**DOI:** 10.3390/s26113333

**Published:** 2026-05-24

**Authors:** Lola El Sahmarany, Maryam Alkhaldi, Saleh I. Alzahrani

**Affiliations:** Biomedical Engineering Department, College of Engineering, Imam Abdulrahman Bin Faisal University, P.O. Box 1982, Dammam 31451, Saudi Arabia; moalkhaldi@iau.edu.sa (M.A.); sialzahrani@iau.edu.sa (S.I.A.)

**Keywords:** driver drowsiness detection, physiological sensors, mechanical sensors, multimodal sensor fusion, biosignal processing, electroencephalography (EEG), electrocardiography (ECG), photoplethysmography (PPG), galvanic skin response (GSR), real-time monitoring systems

## Abstract

Driver drowsiness detection has become an important application of sensor-based monitoring systems aimed at improving road safety. This review focuses on sensing technologies and physiological parameters used for real-time drowsiness detection in drivers. The surveyed approaches are categorized into physiological sensing methods, including electroencephalography (EEG), electrocardiography (ECG), galvanic skin response (GSR), and photoplethysmography (PPG), and mechanical sensing methods, including respiration rate, eye blinking, head movement, yawning, and steering wheel gripping force. Each method is analyzed from a sensor system perspective, considering signal acquisition principles, measurement location, and practical deployment constraints. In addition, the reviewed techniques are evaluated based on real-time capability, level of sensor attachment, cost, restriction of user movement, and suitability for standalone operation. The comparison highlights that mechanical sensing approaches provide non-invasive and cost-effective solutions; however, they are sensitive to environmental noise and behavioral variability. In contrast, physiological sensing methods offer more direct and earlier indicators of fatigue-related changes in biosignals, although they typically require wearable or contact-based sensors and more complex acquisition systems. The review further indicates that multimodal sensor fusion is increasingly being adopted to improve robustness and reliability in real-world driving conditions. Overall, this work provides a structured overview of sensing modalities and highlights key considerations for designing efficient, real-time driver monitoring systems.

## 1. Introduction

Road traffic crashes account for a significant proportion of morbidity and mortality and claim more lives than many diseases. The World Health Organization (WHO) reports that approximately 1.35 million people die annually due to road traffic crashes, and 20 to 50 million people suffer non-fatal injuries, with many experiencing permanent disabilities [1,2]. Road crashes can result from multiple factors, including infrastructure, vehicle conditions, and human variables [3]. Many studies have investigated the primary causes of traffic accidents and have concluded that human factors contribute most significantly to accident severity [3,4,5,6,7].

According to the National Safety Council (NSC), drowsy driving accounts for approximately 100,000 crashes annually, resulting in 71,000 injuries and 1550 fatalities; therefore, it can be considered a major contributor to road accidents, severe injuries, fatalities, and significant economic losses [8]. In general, drowsiness can be defined as a gradual decline in the brain’s processing efficiency; consequently, response time and decision-making ability decrease. Moreover, physiological parameters such as heart rate, body temperature, and respiration rate tend to decrease [9]. In contrast, behavioral features such as yawning and eye blinking tend to increase [10]. Driver impairment caused by factors such as sleepiness, stress, visual inattention, and workload must be detected or predicted to prevent critical situations and crashes.

**Figure 1:**
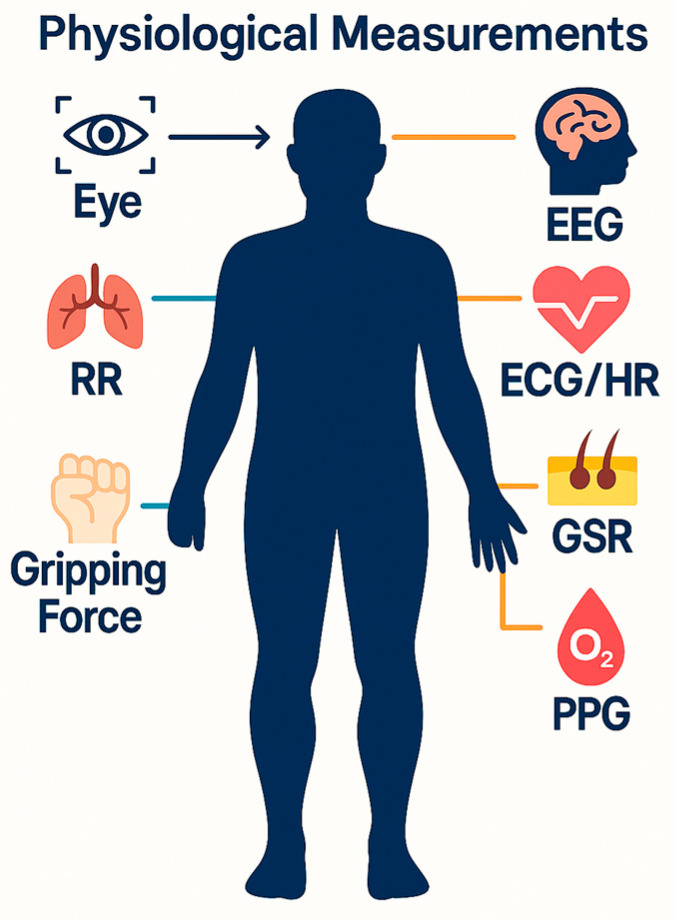
Physiological measurements obtained from the human body.

From a scientific perspective, there is a need to identify suitable indicators for detecting or predicting driver states. These indicators are also essential for evaluating the effectiveness of warning strategies and interfaces once such driver states have been identified. Statistics highlight the need for a reliable driver drowsiness detection system that can alert the driver before a mishap occurs [11]. As a result, several detection techniques have been developed to analyze the behavioral and physiological characteristics of drowsy drivers [12,13,14,15]. The physiological and mechanical measurements considered in this review are illustrated in Figure 1.

Several detection techniques have been developed based on vehicular motion as well as mechanical and physiological phenomena; some of these have been successfully adopted and implemented by leading automotive companies such as Ford, BMW, and Volvo [16]. This paper reviews drowsiness detection techniques that measure drivers’ mechanical parameters, including respiratory rate (RR), gripping force, eye blinking, head movement, eye movement, and yawning. In addition, the physiological parameters considered in this review include electroencephalography (EEG), electrocardiography (ECG), heart rate, galvanic skin response (GSR), and photoplethysmography (PPG).

During drowsiness, the physiological signals of the human body fluctuate. The physiological signals, including heart rate, GSR, and EEG, in both normal and drowsy states are presented in Table 1. Heart rate does not change significantly; however, GSR and EEG exhibit notable variations, making them useful indicators for estimating drowsiness.

## 2. Review Methodology

This review was conducted using a structured narrative approach to provide a comprehensive, technically grounded overview of sensing technologies for real-time driver drowsiness detection. Although the study does not follow a full systematic review or meta-analysis protocol, clearly defined search and selection procedures were employed to ensure transparency, consistency, and methodological rigor.

### 2.1. Literature Sources and Search Strategy

A comprehensive literature search was conducted using major electronic databases covering engineering, biomedical sensing, and intelligent transportation research. Specifically, IEEE Xplore, Scopus, Web of Science, PubMed, ScienceDirect, and MDPI were consulted to ensure broad multidisciplinary coverage.

Search queries were formulated using combinations of keywords related to driver fatigue and sensor-based monitoring, including driver drowsiness detection, driver fatigue monitoring, physiological sensors, EEG-based vigilance detection, ECG and heart rate variability, camera-based driver monitoring, and multimodal driver state detection. Boolean operators (AND/OR) were applied, depending on database syntax, to refine the search results and improve relevance.

### 2.2. Timeframe of the Review

The reviewed literature primarily spans the period from 2000 to 2025, covering both early foundational studies and recent advances in sensor technologies, wearable systems, physiological monitoring, and intelligent driver-monitoring platforms. Earlier studies were selectively included when they provided seminal contributions that established widely accepted concepts or benchmark methodologies, such as early definitions of drowsiness indicators and physiological reference measures. This timeframe enables the review to capture the evolution of driver drowsiness detection from early experimental approaches to modern real-time multimodal systems.

### 2.3. Inclusion Criteria

Articles were considered for inclusion if they met the following criteria:

- Addressed driver drowsiness or fatigue detection in road-driving contexts.
- Employed mechanical, physiological, optical, or multimodal sensor systems.
- Focused on real-time or near-real-time monitoring.
- Were published in peer-reviewed journals or well-established international conference proceedings.
- Written in English.

### 2.4. Exclusion Criteria

Publications were excluded if they:

- Focused exclusively on fatigue in non-driving contexts (e.g., office work, clinical sleep studies without vehicle relevance, or aviation-only studies).
- Did not involve sensor-based data acquisition or analysis.
- Were editorial papers, opinion articles, or conceptual discussions lacking technical or experimental validation.
- Lacked sufficient methodological detail regarding signal acquisition, processing, or system implementation.

### 2.5. Study Selection and Analysis

After the initial search, duplicate records were removed. Titles and abstracts were screened to assess their relevance to driver drowsiness detection, followed by full-text evaluation of potentially eligible articles. The selected studies were analyzed and categorized by sensing modality, measurement location, level of intrusiveness, real-time capability, cost considerations, and suitability for standalone or integrated deployment. This classification framework enabled a structured comparison of mechanical and physiological sensing approaches from both system design and practical implementation perspectives.

### 2.6. Literature Search and Selection Process

**Figure 2:**
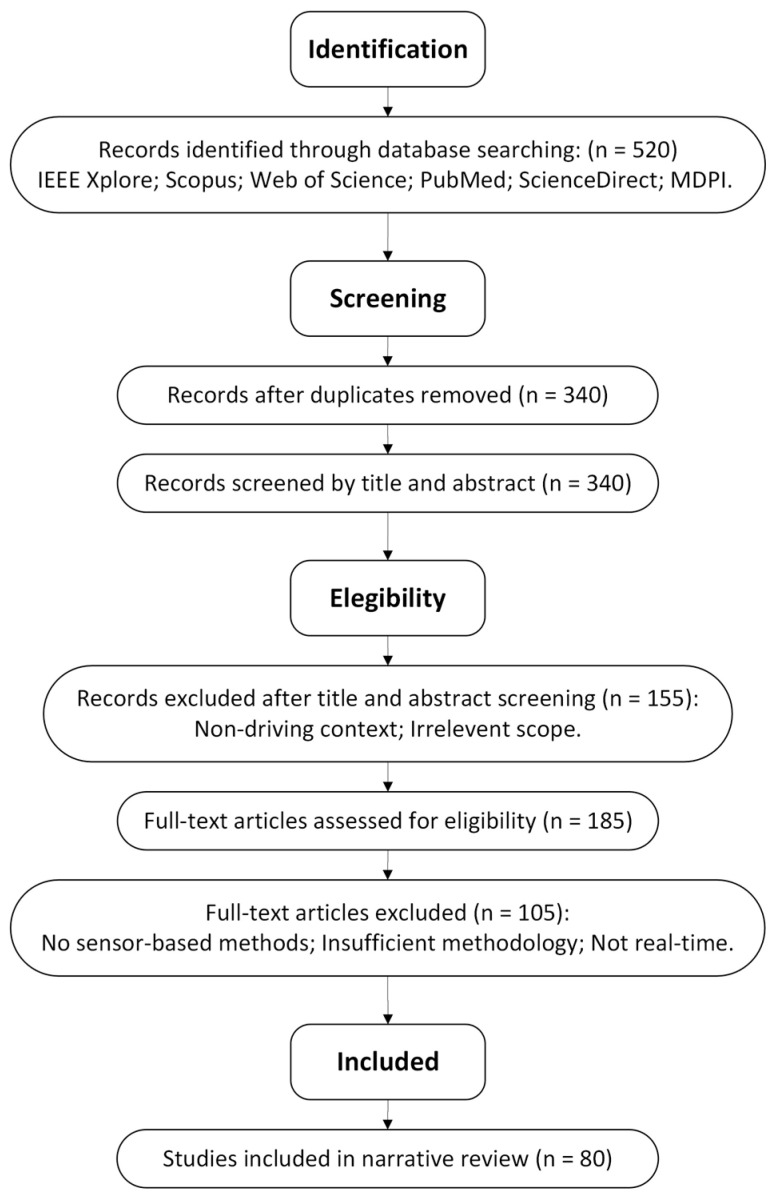
PRISMA-style flow diagram.

To enhance the transparency of the literature search and selection process, a PRISMA-style flow diagram shown in Figure 2 illustrates the identification, screening, eligibility assessment, and final inclusion of studies. Given the narrative nature of this review, the flow diagram is intended to visually summarize the study selection process rather than to represent a formal systematic review or meta-analysis.

## 3. Mechanical Parameters

Mechanical parameters are based on the kinematic variations in the human body. Sensors are used to measure these variations and transmit data for subsequent processing and classification.

### 3.1. Respiratory Rate

The respiratory rate is the number of breaths taken per minute. The usual breathing rate during sleep varies with age. An adult’s normal breathing rate at rest is 12 to 20 times per minute [19]. A strategy for detecting drowsiness based on changes in the respiratory signal was proposed in [20]. The driver’s state was classified as drowsy or awake based on the breathing signal. The signal was acquired using an inductive plethysmography belt and analyzed in real time. The proposed approach uses respiratory rate variability (RRV) analysis to identify physiological changes associated with the transition from wakefulness to drowsiness.

In [21], respiratory rate is obtained using a seatbelt-based system for driver state recognition, where a piezoelectric sensor is integrated to certify a textile cover for a seat belt that includes an optical sensor and a magnetic induction (MI) system. In addition, during trials, it was found that the suggested method improves monitoring of the respiration rate but generates a high-frequency noise signal. In [22], a non-invasive approach was proposed to identify driver drowsiness by capturing breathing rate using two high-dynamic cameras, PAC16 and FRCAM. The cameras were used to record the video data, which was subsequently converted into frames. To regulate lighting conditions in an outdoor environment, histogram equalization was applied to enhance global contrast. Also, to mitigate motion effects during driving, noise filtering and image stabilization were applied. To assess motion levels, frame-differentiation-based approaches were employed, and the image was segmented into regions where motion was detected. After analyzing the motion signals in each segment, non-periodic components were removed. Subsequently, the respiration rate was computed by applying a short-term Fourier transform to the motion signals.

In [22], a system was developed to determine a driver’s level of drowsiness based on spontaneous respiration-related movements captured by cameras. The study investigated robustness across various user types and circumstances. A system consisting of small high-dynamic-range vehicle cameras was presented as a breathing-rate sensing system. The captured images were analyzed to estimate the driver’s chest and abdominal movements. The data were analyzed in real time using a validated algorithm that interprets the detected movement and estimates the driver’s level of fatigue and drowsiness.

The Harken system is a non-intrusive sensing system designed to monitor a driver’s cardiac and respiratory activity through sensors integrated into the car seat cover and seat belt [23]. The system detects the mechanical activity generated by the heart and respiration, filters the acquired signals, and suppresses noise and motion artifacts commonly encountered in moving vehicles. In addition, the system computes relevant physiological parameters and presents them in a suitable format for integration into a fatigue detection system.

A respiratory rate sensor integrated into the seat belt can be used to ensure the driver is wearing the seat belt and to monitor respiratory rate, thereby enhancing driver safety. However, respiratory rate is not widely used for driver drowsiness monitoring because it is highly susceptible to noise and motion artifacts [24]. Table 2 summarizes the techniques based on respiratory rate measurement and their corresponding characteristics.

### 3.2. Eye Blinking

A blink is a rapid opening or closing of one or both eyes. Eye blinking is considered an essential indication of driver drowsiness. Studies have shown that the interval between blinks ranges from 2 to 10 s; under relaxed and normal conditions, a mean blink rate of 10 blinks/min was reported [25,26]. However, eye blinking in drowsy people has been reported to be below 10 blinks/min [26]. Many driver drowsiness detection systems rely solely on this method, while others combine it with additional drowsiness indicators to improve state validation.

Drowsiness detection is primarily performed using a camera positioned to record changes in the driver’s facial behavior, along with corresponding image processing techniques, which will be further discussed in Section 3.3 [27]. Another approach involves placing infrared sensors on or near the driver [28].

When a person focuses on the surroundings, the brain interprets this information, a process known as visual perception. During this process, the ocular motor and attentional systems are active. However, when a person closes their eyes, this process is inhibited, reducing the perception of external visual stimuli. This results in changes in brain activity due to reduced visual stimuli. Eye blinking is a reliable indicator that can be monitored using a sensor module consisting of an eye blink sensor frame and an infrared (IR) sensor. The IR sensor typically includes a transmitter that emits infrared rays toward the driver’s eyes and a receiver that detects the reflected rays when the eyes are closed [29]. This setup enables accurate detection of eye blinks, which can be useful for monitoring driver alertness.

To recognize eye behavior, a real-time system was designed using a video camera and the Viola֪–Jones algorithm, which is widely used for face detection [29]. To determine whether the eye is open or closed, a template-matching method is used to identify eye images that fit a predefined eye-shape template.

In [30], the authors proposed the development and implementation of a lightweight, real-time driver drowsiness detection system for an Android application. The system records video data and detects the driver’s face in each frame using image processing techniques. The system can detect facial landmarks and compute the Eye Aspect Ratio (EAR) and the Eye Closure Ratio (ECR) to assess the driver’s drowsiness using adaptive thresholding. Machine learning algorithms were employed to evaluate the efficacy of the proposed approach. Empirical results demonstrated that the proposed model achieved an accuracy of 84% using a random forest classifier.

Eye blinking provides an instantaneous method for detecting drowsiness; however, blink duration is a critical parameter that must be carefully considered when using this method. The techniques based on eye-blinking analysis and their associated characteristics are summarized in Table 3.

### 3.3. Camera

A camera-based system is an example of an unobtrusive sensing approach suitable for driver applications. Advanced camera-based systems can provide information such as head and gaze direction, eyelid opening, and facial expressions. To operate effectively, the camera system must satisfy automotive requirements, withstand varying lighting conditions, and adapt to different facial features. Camera-based driver drowsiness detection has been adopted in many recent studies, each employing a different processing methodology; however, most approaches rely on analyzing the driver’s facial expressions.

At the Interdisciplinary Graduate School of Science and Technology, Shinshu University, Japan, a drowsiness detection system consisting of a dashboard camera set, an image processing system, and a drowsiness detection model was developed. The system depends on the driver’s facial expressions and information related to the eyes. The proposed method is based on observational analysis, which revealed that features of drowsiness appear on the eyebrows, cheeks, mouth, and eyes [31].

Using facial expressions enables early detection of drowsiness. Features associated with drowsiness were identified by comparing the facial muscle activities of awake and drowsy individuals. Nine facial muscles were monitored during a 1 h monotonous driving task in a driving simulator. The reference drowsiness states were subsequently divided into six levels: not sleepy, slightly sleepy, sleepy, rather sleepy, very sleepy, and asleep.

Subsequently, an image processing method based on the Active Appearance Model (AAM) was developed to detect the 3D coordinates of measurement points on the driver’s face in each frame. Finally, a method for detecting drowsiness level was developed using 17 facial measurement points to identify drowsy expression.

The following study focused on developing a low-cost camera for real-time monitoring. A study conducted at the Department of Computer Science, University of Kerala, India, developed a low-cost camera-based system for real-time monitoring. They used a web camera to monitor the percentage of eye closure (PERCLOS) as a drowsiness parameter [32,33].

The proposed approach relies on detecting open eyes, where the absence of an open-eye pattern indicates that the driver’s eyes are closed. A method called iris–sclera pattern analysis (ISPA) was developed and used to detect open eyes based on the sclera’s axis of symmetry around the iris. Open-eye detection is performed on the local eye region of the face image. The ISPA-based open-eye detection method was incorporated into the PERCLOS approach to facilitate drowsiness detection.

Open-eye detection is continuously performed on the real-time video recorded by the dashboard camera. Detection is performed sequentially on each video frame. The change in face location between two consecutive frames is assumed to be negligible.

In the proposed approach, the PERCLOS system is used to determine the driver’s level of drowsiness. The PERCLOS algorithm calculates the proportion of time that the eyelid covers 80% of the pupil [34]. In this system, a sequence of more than four frames containing closed eyes is considered a PERCLOS state for drowsiness monitoring. The PERCLOS pattern is analyzed every 30 s, where the eye state is identified for each frame; frames classified as PERCLOS state are assigned the value ’one’, while the remaining frames are assigned the value ’zero’.

Camera systems are capable of detecting several indications, such as head position, yawning, and eye blinking. Therefore, this method enables the early detection of drowsiness while reducing the possibility of false alarms. However, advanced camera systems are relatively expensive and may not be suitable for widespread consumer applications. Table 4 presents the evaluation characteristics of the camera-based technique.

Cameras are still among the most popular choices for detecting driver drowsiness, mainly because they can detect eye closure, head orientation, or subtle changes in facial expressions. However, despite their effectiveness, these systems have several limitations. First, they may not perform reliably under poor lighting conditions, such as nighttime driving or strong sunlight causing glare. In addition, glasses, hats, or head movements can obstruct the camera’s field of view. Furthermore, continuous video monitoring may raise privacy concerns among drivers.

To address these limitations, newer contactless technologies have been investigated. For example, some systems use sound waves to monitor respiration by detecting vibrations within the vehicle cabin without requiring external illumination [35]. Others use ultrasound to detect small chest movements, enabling non-contact monitoring of respiration or heartbeat through reflected acoustic waves [36]. Although these methods may provide less behavioral information compared with camera-based systems, they offer several important advantages. They do not require strong lighting conditions, are less intrusive from a privacy perspective, and can operate effectively even when drivers wear sunglasses or heavy clothing. Therefore, as these technologies continue to advance, they may complement camera-based systems or, in certain scenarios, serve as alternative solutions for driver drowsiness detection.

### 3.4. Gripping Force

Gripping force is an economical method for non-invasive and in real-time detection of driver fatigue. A pressure sensor attached to the steering wheel measures the gripping force signal. When a driver becomes drowsy, the gripping force applied to the steering wheel usually decreases as the driver’s muscles relax [37]. Steering-wheel-based measurements can be acquired during both daytime and nighttime driving, making this approach suitable for certain practical driving conditions.

The force-sensitive resistor (FSR) is a thin polymer thick-film resistor (PTR) device that has minimal impact on driving performance. A simple resistance-to-voltage converter circuit converts the resistance to voltage. The force exerted on the wheel is represented by the output voltage V_out_. According to the results reported in [38], males apply significantly greater steering-wheel gripping forces than females. A fatigue detection system using the FSR-408 strip sensor was developed at Chung-Ang University to monitor the grip force [39]. The results showed that grip force decreased significantly as subjective drowsiness increased. Moreover, researchers at Shanghai Jiao Tong University designed a system using two FSRs covering the steering wheel to record grip-force data from both hands [40].

A pair of conductive fabric electrodes can be attached to the steering wheel to monitor ECG signals wirelessly and assess heart rate variability (HRV) as an indicator of drowsiness [41]. Since drowsiness is a complex phenomenon, multiple measurements are often combined to improve the detection of driver fatigue [42].

In [43], an effective real-time monitoring system for drivers’ drowsiness detection was proposed using a gripping force measured by a piezoelectric pressure sensor attached to the steering wheel. The system also detects two additional biological signals: respiration and photoplethysmography (PPG). The studies indicate that variations in steering-wheel gripping force can be utilized to efficiently identify driver drowsiness.

A smart steering wheel was designed to monitor health and drowsiness by integrating ECG and PPG sensors into the steering wheel. An inertial measurement unit was also integrated to provide additional driving movement data alongside the physiological signals, thereby improving the sensory system’s ability to recognize fatigue stages. All collected data are transmitted via Bluetooth to the processor [44].

Grip-force-based detection has been widely investigated in the literature and has been implemented in some commercial vehicles, such as Ford and Mazda. However, this method is often integrated with other methods since the driver may move their hands from the steering wheel for any reason, which makes it unreliable when used as a stand-alone method. The gripping force-based techniques and their corresponding evaluation characteristics are presented in Table 5.

## 4. Physiological Parameters

Physiological signals, on the other hand, begin to change during the early stages of sleepiness. As a result, physiological signals are considered more suitable for detecting drowsiness with fewer false positives, allowing drivers to be alerted in a timely manner and potentially preventing traffic accidents.

### 4.1. Electroencephalogram (EEG)

#### 4.1.1. EEG Electrode and Headset

Electroencephalography (EEG) is the measurement of voltage potential generated by neuronal activity. Electroencephalography (EEG) is the measurement of voltage potential generated by neuronal activity [45]. An EEG recording has five major rhythms: Delta, Theta, Alpha, Beta, and Gamma. For drowsiness detection, delta and theta rhythms are associated with sleepiness and unconsciousness, while alpha rhythms are associated with relaxation. The EEG-based approach is considered an effective and promising method for drowsiness detection [46].

A system using eight Ag-Cl electrodes and two reference electrodes placed near the ear was proposed. The Ag-Cl electrode offers greater applicability than the conventional wet electrode. The OpenBCI (Open Brain–Computer Interface) unit was used to collect, process, and transmit the detected EEG signals. The electrodes were placed based on the International 10–20 system. If the driver was identified as drowsy for 3 s, a warning light was activated. If the driver remained drowsy for more than 5 s, a buzzer was activated while the warning light remained on to regain the driver’s attention. In [47], the driver’s sleep onset was detected using an eight-channel EEG.

With technological advancements, EEG signals can be acquired wirelessly from multiple channels using simpler setups, as EEG electrode placement is time-consuming and drivers generally prefer non-intrusive systems. Emotiv EPOC+ is an EEG device used to acquire neural signals for driver drowsiness detection systems [48]. In [49], driver drowsiness was estimated from the degree of eye closure using four channels of the Emotiv EPOC+ device (P7, O1, O2, and P8), achieving accuracies of 87.5% and 70% for males and females, respectively. In contrast, Ref. [50] used all 14 channels to classify subjects into drowsy or awake states.

A system using Brainsense, a wireless EEG sensor, was proposed to predict sleep onset when the signal reached a predefined voltage threshold [51]. The signal-acquisition module was embedded in a wearable headband device.

In cases of severe drowsiness, alerts alone may not be sufficient to prevent accidents; therefore, additional vehicle control mechanisms may be required. A system using the MindWave Mobile 2 sensor integrated into a helmet was proposed for real-time monitoring [51]. The MindWave Mobile channels were positioned at Fp1, T4, and A1 according to the international 10–20 system. The driver is alerted by an alarm, and the motor slows down and stops. The motor and alarm were controlled using Arduino-based software.

#### 4.1.2. In-Ear EEG

Wearable EEG electrodes are used to monitor brain activity in hospitals and to assess driver vigilance in vehicles. Therefore, the development of in-ear EEG technology has enabled more unobtrusive EEG acquisition. Wearable in-ear devices are widely accepted because they impose minimal limitations on daily activities. Ear-EEG technology has recently been proposed for sleep monitoring, as in-ear systems can significantly reduce the complexity of conventional bulky setups.

The recent development of hearing aids incorporating bioelectrical sensors suggests that the ear may be a promising site for physiological monitoring [52]. In addition, in-ear measurements can detect multimodal physiological signals [53]. The sensor consists of a viscoelastic memory-foam substrate placed in the ear canal with a microphone attached beneath the cloth electrodes. The proposed design enables monitoring of brain, cardiac, and respiratory activity.

Another system further demonstrated the feasibility of in-ear EEG drowsiness monitoring by comparing the accuracy of scalp EEG and in-ear EEG using a viscoelastic sensor [54]. The sensor is designed based on ‘one-size-fits-all,’ and because viscoelastic materials absorb energy, the earpiece can reduce the impact of motion artifacts, especially those caused by pulsatile motions of the ear canal wall. More details about its mechanical and electrical characteristics can be found in [55]. Scalp EEG achieved an accuracy range from 86.8% to 88.8%, while in-ear achieved a range from 80.0% to 82.9% [53].

EEG is considered one of the most important sources of data for effective drowsiness detection [45,55]. In general, increasing the number of sensing channels improves classification accuracy. Also, channel selection is a critical factor. Several studies have used O1 and/or O2 channels to detect drowsiness with accuracies reaching 93.87% [56,57]. Drivers generally prefer wearable EEG helmet devices over conventional gel-based EEG electrode setups, such as Brainsense, MindWave mobile, and Emotive EPCO+. In-ear EEG sensors have continued to evolve to provide consistent waveform acquisition while maintaining comfort during long-term monitoring [58]. The evaluation characteristics of EEG-based drowsiness detection techniques are summarized in Table 6.

### 4.2. Galvanic Skin Response (GSR)

The skin covers the entire body and is considered the largest organ of the human body. It serves as a protective barrier for the body’s internal structures and regulates body temperature through sweating [59]. The skin consists of three main layers, the epidermis, dermis, and hypodermis, each containing structures that contribute to its electrical and physiological properties. The thickest areas are found on the hand palm, foot sole, and buttocks. In contrast, the thinnest areas are found on the eyelids [60].

Galvanic skin response (GSR), also known as electrodermal activity (EDA), refers to autonomic changes in the skin’s electrical properties. It is most commonly measured as skin conductance (SC) [61]. Electrodermal activity is a physiological signal that is typically measured noninvasively with electrodes placed on the skin surface.

Applying a potential difference between two sites of the skin enables the observation of current flow through it. This is due to the movement of free ions present in the skin structures. Sweat ducts, blood, and intestinal fluids have different ionic concentrations and therefore have different conductivities. Bloodstream, lymph, and interstitial fluids make the skin dermis a good conductor [61]. Skin conductance is one of the physiological parameters used to detect emotional arousal. It also measures physiological reactions such as fear and stress.

Human skin can disclose a great deal about how a person is feeling at any given moment. Various pieces of information about the vigilance state of the driver, including fatigue and drowsiness, can be obtained through GSR. More importantly, the GSR phasic component is significantly affected by hand gripping force as well as eyelid closure, which are both indications of the drivers’ fatigue and drowsiness [62]. Due to the significant changes in GSR caused by handgrip and eye state, this method is useful for monitoring driver drowsiness.

In 2015, at the Poornima College of Engineering in India, engineers implemented a basic k-means classifier algorithm for modification of the dataset of skin conductance (SC) signals. The k-means algorithm is a clustering method used to group datasets obtained from signal processing. An important aspect of this method is that the test results achieved 100% classification accuracy [63].

In 2017, Ford Motor Company conducted a project in cooperation with the iMotions platform for human research and the Mindshare global media agency. In the study, galvanic skin response was used in conjunction with electroencephalograms and facial expressions. The experiment was performed using biosensors and a video camera with different participants, and the data were recorded. The output GSR signal was then analyzed to calculate the threshold and peak amplitude for detecting driver drowsiness [64].

In 2020, the STEER wearable device was created by the Creative Mode design studio. The device analyzed the driver’s skin conductance and heart rate every two seconds and warned the driver through vibration and a gentle electrical impulse when these values decreased, thereby helping to prevent drowsiness while driving [65].

The evaluation characteristics of techniques employing galvanic skin response are presented in Table 7.

### 4.3. Photoplethysmography (PPG)

Photoplethysmography (PPG) is a simple non-invasive optical method used to detect and measure pulse wave, blood volume, and blood oxygen saturation (SpO_2_) in a microvascular tissue [66].

Normal blood oxygen saturation is typically 95% or higher; however, values starting from 90% may be considered normal for some individuals with chronic lung diseases [67].

This method has been used to support driver drowsiness detection systems based on a reduction in oxygen during sleep due to decreased breathing activity. Generally, the measurements of this method are obtained via an oximetry process in which a small clip-on device is used to emit light through the finger or earlobe; typically, red and infrared wavelengths of 660 and 940 nm, respectively, are used. The amount of light absorbed by the oxygen-carrying hemoglobin in red blood cells is then measured to determine the hemoglobin oxygen saturation [68].

A driver monitoring system designed by Keimyung University in Korea used a PPG sensor in combination with other tools to monitor the driver’s biological signals in real time. PPG signals were measured using a sensor consisting of a light-emitting diode (LED) and a phototransistor attached to the steering wheel. The obtained signals were then processed to calculate heart rate (HR) and heart rate variability (HRV) using a system developed in National Instruments LabVIEW software [69].

In 2019, a collaboration between the University of North Dakota and Jilin Normal University resulted in the development of a drowsiness detection system investigating pulse arrival time (PAT) and PPG features as indicators of drowsiness. Simultaneous vertical electrooculogram (EOG), ear PPG, and electrocardiogram (ECG) were recorded from pilots. ECG R-peaks and PPG peaks were detected and used for the calculation of PAT and heart rate to observe their changes during drowsiness. It was concluded that PAT and derived PPG-based features, combined with continuous heart rate monitoring, can serve as useful indicators for early drowsiness detection [70].

Another detection system was developed at Kyungpook National University to analyze heart rate variability (HRV) signals acquired from wearable electrocardiogram (ECG) or photoplethysmogram (PPG) sensors. Since wearable sensors are sensitive to slight motion, reliable features are needed to distinguish between drowsy and awake states in noisy HRV signals. Three types of recurrence plots (RPs) generated from the R–R intervals (RRIs) of heartbeats were explored: Binary recurrence plot (Bin-RP), continuous recurrence plot (Cont-RP), and thresholded recurrence plot (ReLU-RP), obtained by filtering Cont-RP using a modified rectified linear unit (ReLU) function. By utilizing each of these RPs as input features to a convolutional neural network (CNN), their usefulness for drowsy/awake classification was examined. For experiments, RRIs under drowsy and awake conditions were collected with an ECG sensor of the Polar H7 strap and a PPG sensor of the Microsoft (MS) band 2 in a virtual driving environment. The results showed that ReLU-RP was the most distinct and reliable pattern for drowsiness detection, regardless of sensor types (i.e., ECG or PPG). In particular, the ReLU-RP-based CNN models showed their superiority to other conventional models, providing approximately 6–17% better accuracy for ECG and 4–14% for PPG in drowsy/awake classification [71].

The PPG method is most commonly used in combination with other methods as a supporting parameter, since it produces only minor changes that may be insufficient for detecting drowsiness independently. In addition, PPG signals can be affected by motion and ambient light. The PPG-based techniques and their corresponding evaluation characteristics are summarized in Table 8.

### 4.4. Electrocardiogram (ECG) & Heart Rate

ECG represents the electrical signals produced by the heart during each heartbeat. For ECG measurements, many types of electrodes can be utilized, each attached to a specific scanning location. As a result, several studies have focused on implementing ECG systems in vehicles for drowsiness detection. The heart rate (HR) is the number of heartbeats in a particular time unit. As a result, heart rate, which is easily determined by an ECG signal, can be used to detect drowsiness [72].

The electrocardiogram (ECG) is a signal representing the electrical activity of the heart measured from a specific location on the human body. In [73], linear regression was used to segment the ECG signal for detecting the R point and subsequently identifying the P, Q, S, and T peaks. The method achieved a sensitivity of 99.5% to identify all P, Q, R, S, and T peaks.

Since heart rate (HR) can be easily calculated from the ECG signal, it has been used for drowsiness detection. In [74], drowsiness was measured using heart rate variability (HRV), where the low-frequency (LF) and high-frequency (HF) bands were 0.04–0.15 Hz and 0.14–0.4 Hz, respectively, using a support vector machine (SVM) for data classification. The method achieved a mean accuracy of approximately 58–59%. Additionally, a system was developed to identify early-stage driver drowsiness using a logistic regression-based machine learning method to compute heart rate variation, achieving an accuracy of over 92% [30].

In [75], a method for detecting drowsiness using combined EEG and ECG data was presented. The suggested method is based on a convolutional neural network, a recurrent neural network, and a deep learning architecture. The proposed method achieved accuracy scores of up to 97% on the validation set. The study also showed that incorporating autoencoders into the proposed design helped compensate for performance reduction when analyzing subjects whose data were not included during the training stage.

ECG and HR parameters may change during drowsiness and can provide useful indicators when measured accurately. However, these methods are not always considered reliable sleepiness indicators because they are susceptible to the effects of physical activity, stress, and the emotional state of the driver. Table 9 provides an overview of ECG-based drowsiness detection techniques along with their evaluation characteristics.

### 4.5. Temperature

Body and skin temperature are widely recognized as reliable physiological biomarkers for fatigue assessment due to their sensitivity to metabolic demands and thermoregulatory stress. According to [76], continuous temperature monitoring using wearable sensors has shown excellent accuracy and robustness in capturing physiological strain under real-world conditions during prolonged task performance. Temperature is a useful and non-invasive indicator for fatigue-related risk monitoring because fatigue is frequently associated with impaired thermoregulation, such as gradual increases in skin temperature due to decreased autonomic responsiveness. Furthermore, the integration of temperature-based wearable devices into multimodal fatigue detection systems is supported by their practicality and comfort in field studies.

## 5. Future Perspectives and Emerging Non-Invasive Optical Monitoring Technologies

Beyond traditional mechanical and physiological sensors, emerging non-invasive optical monitoring methods show significant potential for driver drowsiness detection. Recent advances in optical imaging enable the detection of even slight changes inside the body, including variations in biomarkers associated with fatigue. Monte Carlo simulations can be used to model light propagation through biological tissues, facilitating the design of sensors that work well with techniques such as near-infrared spectroscopy. These developments may enable future driver monitoring systems to incorporate comfortable, wearable, or even contactless sensors, paired with smart computer models, to detect signs of drowsiness earlier and more reliably. Although these optical technologies are mostly still in laboratory and clinical environments, ongoing advances in miniaturization and usability may facilitate their integration into vehicles as practical, non-intrusive safety tools [77,78,79].

## 6. Comparative Overview of Reviewed Techniques

Table 10 summarizes the main characteristics of the reviewed drowsiness detection techniques, including their measured parameters, sensor locations, advantages, and limitations. Comparative analysis of the reviewed techniques indicates that each approach offers distinct advantages and limitations. Physiological sensors such as EEG and ECG provide high accuracy for drowsiness detection. However, they may cause discomfort for drivers and often require complex setup procedures, making them less practical for daily use. In contrast, behavioral monitoring methods, such as eye blink detection and camera-based facial expression analysis, are less intrusive and provide rapid feedback, but their effectiveness can drop if the lighting is not right or if the sensors are not placed perfectly. Simpler methods, such as steering-wheel grip force measurement and heart rate monitoring, are easy to use and cost-effective; however, their measurements may be influenced by factors not related to drowsiness. The selection of an appropriate detection method relies on the requirements of the situation, whether it is accuracy, comfort, budget constraints, or ease of use. Future integration of several of these approaches could be the key to building systems that are both reliable and user-friendly.

## 7. Conclusions

This review paper mainly focuses on driver drowsiness detection techniques, which are divided into two categories, mechanical and physiological, based on the measured parameters. When designing and evaluating such systems, several factors must be considered, including real-time capability, minimal sensor attachment, cost-effectiveness, unrestricted driver movement, and the system’s ability to independently and accurately detect drowsiness.

Real-time monitoring is essential, as immediate detection and alerting are necessary to ensure driver safety. To minimize interference with driving activity, effective systems aim to reduce the need for direct sensor attachment and avoid restricting the driver’s movements. Cost is also an important consideration for widespread adoption.

Since drowsiness is a complex phenomenon not yet fully understood, some methods may not be sufficient to immediately recognize the drowsy state as it occurs. As a result, some detection systems combine multiple methods to improve the reliability of the system’s response; however, certain methods are sufficiently robust to be implemented independently and still provide accurate results.

## Figures and Tables

**Table 1:** Physiological signal values in normal and drowsy states.

Physiological Signals	Normal State	Drowsy State
Heart rate [16]	89.8 ± 5.6 bpm	81.5 ± 9.2 bpm
GSR [17]	2–20 µS	<2 µS
Eye blink duration [18]	0.1–0.4 s	0.5–0.65 s

**Table 2:** Techniques and their characteristics related to respiratory monitoring methods.

Techniques/Evaluation Characteristics	Seatbelt	Camera
Real-Time	Yes	Yes
Attached	Yes	No
Cost	Medium	High
Restriction of Driver Movement	Low	Low
Stand-Alone	Low	High

**Table 3:** Techniques and their characteristics related to eye blinking methods.

Techniques/Evaluation Characteristics	Camera	Infra-Red
Real-Time	Yes	Yes
Attached	No	Yes
Cost	High	Medium
Restriction of Driver Movement	Low	High
Stand-Alone	High	High

**Table 4:** Characteristics of the camera-based method.

Techniques/Evaluation Characteristics	Camera
Real-Time	Yes
Attached	No
Cost	High
Restriction of Driver Movement	Low
Stand-Alone	High

**Table 5:** Characteristics of the gripping force-based method.

Techniques/Evaluation Characteristics	Force Sensitive Resistor (FSR)
Real-Time	Yes
Attached	Yes
Cost	Low
Restriction of Driver Movement	Low
Stand-Alone	Low

**Table 6:** Techniques and their characteristics related to EEG-based method.

Techniques/Evaluation Characteristics	Headset	In-Ear
Real-Time	Yes	Yes
Attached	Yes	Yes
Cost	High	Low
Restriction of Driver Movement	High	Medium
Stand-Alone	High	Medium

**Table 7:** Characteristics of the galvanic skin response (GSR)-based method.

Techniques/Evaluation Characteristics	Galvanic Skin Response (GSR)
Real-Time	Yes
Attached	Yes
Cost	Medium
Restriction of Driver Movement	Medium
Stand-Alone	High

**Table 8:** Characteristics of the pulse oximetry-based PPG method.

Techniques/Evaluation Characteristics	Pulse Oximetry
Real-Time	Yes
Attached	Yes
Cost	Low
Restriction of Driver Movement	High
Stand-Alone	Low

**Table 9:** Characteristics of the ECG seatbelt-based method.

Techniques/Evaluation Characteristics	ECG Seatbelt
Real-Time	Yes
Attached	Yes
Cost	Low
Restriction of Driver Movement	Low
Stand-Alone	Low

**Table 10:** Summary of the Reviewed Drowsiness Detection Techniques.

	Measured Parameter	Location Site	Advantages	Limitations
Respiration rate	Breathing rate	Chest	Easy to implement	Sensitive to movement artifact.
Eye Blinking	Eye closure	Limited distance from eye	Fast response	Placement of the sensor is critical
Video Camera	Facial expression	Dashboard camera set	Non-intrusive, instant response	Expensive, require high processing capacity, sensitive to environment.
Gripping Force	Pressure	Hand grip	Economical	Delayed response
EEG	Brain activity	Scalp	Good Robustness	Intrusive, complicated setup
GSR	Skin conductance	Palm/sole	Non-intrusive, fast response	Needs steady direct contact
PPG	Arterial oxygen saturation	Tip of the finger/ear	Continuous monitoring	Affected by ambient light.
ECG	Heart activity	Chest	Accurate indication	Sensitive to movement artifact.
Heart Rate	Beats rate	Chest/wrist	Easy to implement	Variation depending on the driver’s emotional state.

## Data Availability

No new data were created or analyzed in this study.

## Abbreviations

The following abbreviations are used in this manuscript:

CNN	Convolutional Neural Network
ECG	Electrocardiogram
EDA	Electrodermal Activity
EEG	Electroencephalogram
EOG	Electrooculogram
FSR	Force Sensitive Resistor
GSR	Galvanic Skin Response
HR	Heart Rate
HRV	Heart Rate Variability
IR	Infrared
ISPA	Iris–Sclera Pattern Analysis
PAT	Pulse Arrival Time
PERCLOS	Percentage of Eye Closure
PPG	Photoplethysmography
ReLU	Rectified Linear Unit
ReLU-RP	Rectified Linear Unit–Recurrence Plot
SC	Skin Conductance
